# *In-Vivo* Nonlinear Optical Microscopy (NLOM) of Epithelial-Connective Tissue Interface (ECTI) Reveals Quantitative Measures of Neoplasia in Hamster Oral Mucosa

**DOI:** 10.1371/journal.pone.0116754

**Published:** 2015-01-29

**Authors:** Rahul Pal, Jinping Yang, Daniel Ortiz, Suimin Qiu, Vicente Resto, Susan McCammon, Gracie Vargas

**Affiliations:** 1 Center for Biomedical Engineering, The University of Texas Medical Branch, Galveston, TX, 77555, United States of America; 2 Department of Radiology, Eastern Virginia Medical School, Norfolk, VA, 23507, United States of America; 3 Department of Otolaryngology, The University of Texas Medical Branch, Galveston, TX, 77555, United States of America; 4 Department of Pathology, The University of Texas Medical Branch, Galveston, TX, 77555, United States of America; 5 Department of Neuroscience and Cell Biology, The University of Texas Medical Branch, Galveston, TX, 77555, United States of America; 6 Department of Biochemistry and Molecular Biology, The University of Texas Medical Branch, Galveston, TX, 77555, United States of America; 7 Center for Cancers of the Head and Neck, The University of Texas Medical Branch, Galveston, TX, 77555, United States of America; University of California, Merced, UNITED STATES

## Abstract

The epithelial-connective tissue interface (ECTI) plays an integral role in epithelial neoplasia, including oral squamous cell carcinoma (OSCC). This interface undergoes significant alterations due to hyperproliferating epithelium that supports the transformation of normal epithelium to precancers and cancer. We present a method based on nonlinear optical microscopy to directly assess the ECTI and quantify dysplastic alterations using a hamster model for oral carcinogenesis. Neoplastic and non-neoplastic normal mucosa were imaged *in-vivo* by both multiphoton autofluorescence microscopy (MPAM) and second harmonic generation microscopy (SHGM) to obtain cross-sectional reconstructions of the oral epithelium and lamina propria. Imaged sites were biopsied and processed for histopathological grading and measurement of ECTI parameters. An ECTI shape parameter was calculated based on deviation from the linear geometry (ΔLinearity) seen in normal mucosa was measured using MPAM-SHGM and histology. The ECTI was readily visible in MPAM-SHGM and quantitative shape analysis showed ECTI deformation in dysplasia but not in normal mucosa. ΔLinearity was significantly (p < 0.01) higher in dysplasia (0.41±0.24) than normal (0.11±0.04) as measured in MPAM-SHGM and results were confirmed in histology which showed similar trends in ΔLinearity. Increase in ΔLinearity was also statistically significant for different grades of dysplasia. *In-vivo* ΔLinearity measurement alone from microscopy discriminated dysplasia from normal tissue with 87.9% sensitivity and 97.6% specificity, while calculations from histology provided 96.4% sensitivity and 85.7% specificity. Among other quantifiable architectural changes, a progressive statistically significant increase in epithelial thickness was seen with increasing grade of dysplasia. MPAM-SHGM provides new noninvasive ways for direct characterization of ECTI which may be used in preclinical studies to investigate the role of this interface in early transformation. Further development of the method may also lead to new diagnostic approaches to differentiate non-neoplastic tissue from precancers and neoplasia, possibly with other cellular and layer based indicators of abnormality.

## Introduction

The junction between the epithelium and underlying connective tissue, also known as the epithelial-connective tissue interface (ECTI), is an important site in epithelial carcinogenesis as it represents the site of the basement membrane where communication between epithelium and lamina propria occur [1]. It is the progression of neoplastic cells from the epithelium across this interface that defines transformation to invasive carcinoma and presents the potential for metastasis. Prior to invasion, however when neoplasia is histologically confined to the epithelium as in the case of dysplasia including high-grade dysplasia or carcinoma-in-situ, cellular abnormalities including genomic instability, hyperproliferation and loss of cell polarity are present as are subepithelial matrix abnormalities evident by remodeling of the extracellular matrix (ECM) architecture [2], the latter recognized as a crucial hallmark of carcinogenesis [3,4]. Interactions between neoplastic cells, the basement membrane (BM) and ECM are believed to give rise to alterations in the structure of the ECTI [5]. These alterations can be due to mechanical stresses from growing epithelium as well as upregulation of proteases such as matrix metalloproteinases [6–8]. In neoplasia the deregulation of ECM dynamics is initiated by the action of matrix metalloproteinases (MMPs) which are activated by growth factors and cytokines from hyper proliferative dysplastic cells in the epithelium lying just above the ECTI [9–11]. Additionally, hyper-proliferative epithelial cells in the neoplastic foci induces thickening of the epithelium, which applies “compression stress” [6] and may contribute to deformation of the ECTI. These types of deregulation of ECM dynamics are suggested to alter matrix stiffness and induce pockets of matrix compliance [6].

Since the ECTI and the processes leading to alterations in ECTI morphology are integral to carcinogenesis, much regarding the process of neoplastic transformation could be learned from methods that allow direct and noninvasive study of this key interface. Such a capability could be useful in the enhanced understanding of early events of epithelial neoplasia such as identification of patterns associated with pre-invasion and early invasion stages. This may also provide important image markers that could be used to develop additional *in-vivo* metrics to identify sites of dysplasia. Although histological evaluation is the current gold standard for ECTI assessment, it requires biopsy, tissue fixation and sectioning, and does not allow noninvasive visualization or study [12,13]. Other methods of imaging, such as electron microscopy, have required both fixation and removal of the epithelium to visualize the ECTI and abnormalities in structure which accompany precancer [14,15]. The method of optical coherence tomography (OCT), with axial resolution range of 7–17 μm has been used to obtain cross-sectional optical images to encompass both the epithelium and lamina propria in a noninvasive manner and has been investigated in the context of oral dysplasia in several studies [16–21]. Two of these studies have used advanced segmentation algorithms to delineate the oral epithelium-lamina propria boundary demonstrating the potential for extraction of ECTI features using this modality [20,21], however no known OCT studies have extracted ECTI-specific parameters or made direct quantitative comparisons with histology to validate ECTI shape features.

In this study we apply nonlinear optical microscopy (NLOM) by the modalities of multiphoton autofluorescence microscopy (MPAM) and second harmonic generation microscopy (SHGM) to image the ECTI as these imaging modalities are capable of performing deep tissue *in-vivo* imaging at high resolution (~1.7 μm axial) enabling layer-resolved imaging of tissue morphology at subcellular level. Autofluorescence in label-free MPAM is typically used for morphometric and biochemical analysis of intact tissue [22–26]. SHG microscopy can be used to image ECM components with noncentrosymmetric molecular structure comprising of fibrillar collagen [27–29]. Together, these two imaging modalities can provide information about the epithelium (cellular features, thickness) as well as the underlying lamina propria architecture on a microscopic level. Further we believe they can be used to specifically delineate the ECTI and features associated with neoplasia that are unique to that interface. The ability to delineate and quantify parameters of ECTI would be useful in studies investigating the interplay between epithelium and lamina propria and examination of alterations relative to each other in neoplasia. Because the BM refers specifically to the layer consisting of Type IV collagen separating the epithelium and lamina propria and itself is not necessarily distinct in MPAM-SHGM, we use the term ECTI to describe the uppermost lamina propria interface lining the BM visible by SHGM.

The purpose of this study was to evaluate whether changes in the ECTI which occur with dysplasia are visible by MPAM-SHGM and to develop a quantitative approach to describe these early changes using a hamster model of oral carcinogenesis for establishing the concept. For reasons outlined above, alterations in the shape of the ECTI were expected to correlate to dysplasia, thus SHGM cross sectional images from normal and dysplastic oral mucosa were analyzed. Results from a semi-automated image analysis approach indicate ability to identify and quantify features in ECTI associated with dysplasia while more traditional measures of cellular and layer based abnormalities remain possible with these imaging modalities.

## Materials and Methods

### Animal Model

**Fig 1:**
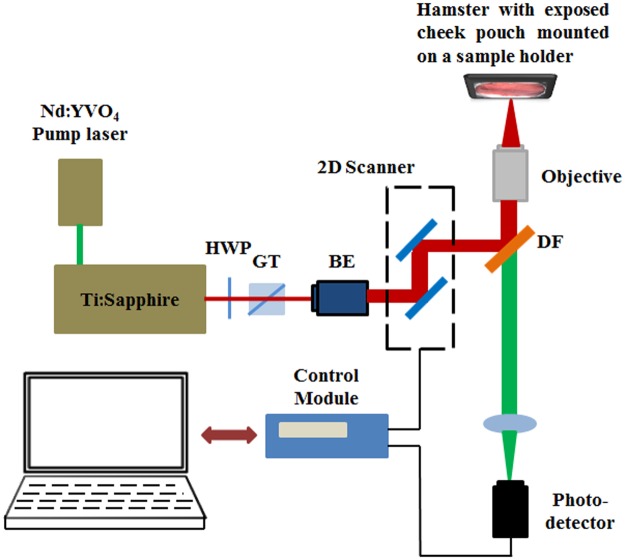
Schematic describing the experimental setup for *in-vivo* multiphoton autofluorescence microscopy (MPAM) and second harmonic generation microscopy (SHGM). HWP: half wave plate; GT: Glans Thompson prism; BE: beam expander; DF: dichroic filter. The hamster is placed on the stage of the inverted microscope with the buccal pouch exposed and facing the objective.

A Golden Syrian Hamster buccal model of dysplasia and oral cancer involving topical application of 9,10-dimethyl-1,2-benzanthracene (DMBA) was used [30–36]. Histologically hamster buccal mucosa shows very similar features to sites of the human oral cavity including the floor of the mouth and ventral surface of the tongue, common sites for neoplastic transformation. The DMBA model of oral carcinogenesis is a well-characterized model that shows histological similarities to human oral precancer and cancer, following a similar multistep progression from normal to increasing grades of dysplasia and carcinoma-in-situ to cancer [30–36]. Additionally, studies have examined the multistep process involving genetic events such as deregulation of oncogenes and tumor suppressor genes in this model [33] and shown parallels in molecular marker alterations (e.g. p53, c-myc, p16, Bcl-2 etc.) to human [35,36]. The well documented histological and molecular similarities between this model and human oral precancer/cancer make this a useful model to study morphometric features associated with neoplasia. For this study, oral dysplasia was induced by topical application of 0.5% DMBA in mineral oil on the left cheek pouch three times a week for 8 weeks (n = 21). Additional hamsters were treated only with mineral oil and used as controls (n = 5). After 8 weeks of DMBA treatment hamsters were anesthetized with a mixture of 150-mg/kg ketamine and 2.5-mg/kg xylazine via intraperitoneal injection. The cheek pouch, a loose pouch of buccal mucosal tissue was stretched outside of the oral cavity and attached to a sample holder having a flat surface. The pouch was rinsed with sterile PBS at room temperature and the hamster placed on a sample holder for microscopy with the buccal pouch accessible to the imaging objective (Fig. 1). After MPAM-SHGM imaging the animals were sacrificed and biopsies were obtained from each imaged site and fixed in 10% neutral-buffered formalin. The fixed samples were embedded in paraffin, sectioned and stained by hematoxylin and eosin (H&E) for histopathological grading. Animal studies were approved by the Institutional Animal Care and Use Committee at the University of Texas Medical Branch and conformed to the Guide for the Care and Use of Laboratory Animals.

### Microscopy

Multiphoton autofluorescence microscopy (MPAM) and second harmonic generation microscopy (SHGM) were performed using a custom built microscopy system, described previously [37], that used a Ti:Sapphire femtosecond (~100fs, 82MHz) laser (Tsunami, Spectra Physics, Mountainview CA), tunable within the wavelength range of 700–1000 nm. Fig. 1 depicts a schematic of the experimental setup, based on an inverted microscope configuration with a sample holder supporting the hamster such that the cheek pouch surface faced the objective. Second harmonic generation originating from the collagen I rich lamina propria was imaged using 840 nm illumination through a 40x, 1.2 N.A. water immersion objective (C-Apochromat), and collected through a 420/20 nm bandpass filter. MPAM to visualize the keratinizing stratum corneum, epithelium and lamina propria by autofluorescence was obtained using 780 nm laser illumination and a broadband emission filter encompassing wavelengths 450–650 nm. Detection was done by cooled PMTs (R6060, Hamamatsu, Japan). The average illumination power at the sample was maintained at 25mW using intensity control from a quarter wave plate and Glans-Thompson polarizer combination. A 10X, 0.3 NA air objective (Plan-Neofluar) was used to first localize regions of interests (ROIs) and the 40x, 1.2 N.A. objective was used for high resolution microscopy. Grayscale images (8-bit) were acquired with 512x512 pixels and 0.625μm/pixel encompassing a 320x320 μm field-of-view (FOV) while image stacks were acquired with z-steps of 1μm. Cross-sections of the ROIs were also imaged with a built in z-scanning function in the image acquisition software with the site for acquisition chosen at the center, in the case of a lesion visible by microscopy.

H&E stained samples from sites imaged *in-vivo* by MPAM-SHGM were imaged with an Olympus IX71 inverted brightfield microscope using a 20X, 0.75 NA air objective. Samples were graded in four categories (normal, mild dysplasia, moderate dysplasia, severe dysplasia) and sites representing the most central region having the most severe pathological features were selected for quantitative measurements. Grading was performed according to WHO’s criteria for architectural and cytological changes in dysplastic epithelium [38]. Two sections closest to the center of lesions encompassing approximately 600μm (twice the FOV of imaging) were chosen for measurements. This was to maintain consistency with the imaging protocol in which the most central region of a lesion was imaged.

### Image Processing and Data Analysis

**Fig 2:**
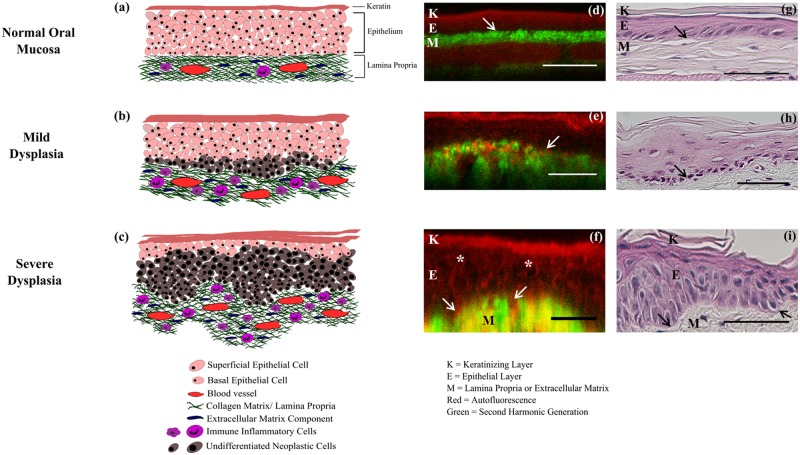
Early events during neoplastic transformation. Schematic representation of the current understanding of cellular and layer-based changes with neoplasia (a-c), MPAM-SHGM micrographs (d-f) and histological sections (g-i) of hamster oral mucosa for normal (a, d, g), mild dysplasia (b, e, h) and severe dysplasia (c, f, i) are shown. *In-vivo* autofluorescence from MPAM (Red) co-registered with SHG (Green) are overlaid to produce cross-sectional MPAM-SHGM micrographs (d-f) comparable to histology (g-i). K: Keratinizing layer; E: Epithelial layer, M: Lamina propria or ECM. White and black arrows point towards ECTI and “*” shows enlarged nuclei in dysplastic epithelium. Scale bar: 50μm.

Cross-sectional x-z/y-z views of the image stacks were obtained using Metamorph (Molecular Devices, Sunnyvale, CA). Cross-sections from MPAM and SHGM of each imaged site were overlaid (Fig. 2d-f) for visualization of the distinct separation of the epithelium, lamina propria and ECTI. The thickness of the epithelium was measured from MPAM-SHGM and histology cross-sectional images by calculating the distance between the surface of the epithelium and the ECTI using a line tool in ImageJ (NIH). Four separate measurements were made for each site and average calculated. The ECTI surface was initially identified from co-registered MPAM-SHGM images [24] as the topmost border in SHGM cross-sectional images. These images were thresholded and an edge detection algorithm plugin was applied to extract the ECTI (“FeatureJ: Edges” plugin in ImageJ). Consistent with known alterations in structure [22,39], qualitative observation indicated deviation from a flat surface in cases of dysplasia, resulting in areas where the ECTI curved deeper into the tissue. The expected flat surface of the ECTI in normal buccal mucosa was modeled by a straight line across the cross-sectional image, and the degree of deviation from a flat surface was modeled as a curved surface which deviated in shape from a straight line. A quantitative measure of this deviation was devised and applied to SHGM cross-sections. Mapping of the ECTI and calculation of its length (*l*) was performed using a “simple neurite tracer” plugin [40], also in ImageJ. The measured curved length was then normalized with a theoretical ECTI representing a straight line (*L*) connecting the two ends of the image within the same field-of-view.

The normalized deviation of ECTI from linearity, i.e. ΔLinearity (*ΔL*_*norm*_) was calculated using the following equations;

ΔL=LengthofECTI(l)−straightlineECTI(L)(ΔL)norm=ΔLL

Statistical comparison of *(ΔL)*_*norm*_ and epithelial thickness among normal and different grades of dysplasia was achieved by single factor ANOVA followed by Tukey’s *post hoc* test. SPSS software was used to generate a receiver operating characteristic (ROC) curve for *(ΔL)*_*norm*_ to separate normal from dysplasia. Area under the curve (AUC) was also measured in SPSS from ROC curves. True positive/negative and false positive/negative values for *(ΔL)*_*norm*_ were calculated from ROC curves for the optimum sensitivity and specificity.

## Results

Fig. 2 shows a process believed to lead to alterations in the epithelium and ECTI morphology during dysplastic transformation [41] shown alongside MPAM-SHGM images and histology from the current study. Fig. 2a-c is a representation of the expected alterations in tissue architecture from normal (Fig. 2a) to mild dysplasia (Fig. 2b) to severe dysplasia (Fig. 2c). The topmost layer is composed of terminally differentiated keratinocytes forming the superficial stratum corneum surface. The underlying epithelium is divided into superficial and basal epithelial layers. Cell division takes place primarily in the basal cell layer, with basal cells undergoing a differentiation process and moving superficially to form the stratum spinosum and finally stratum corneum before being sloughed off the surface [42–44]. The ECM/ lamina propria is primarily composed of collagen, elastin, and laminin networks and act as a supporting matrix for the epithelium. In the current understanding, neoplastic transformation begins in the basal epithelium [45–48]; increased and uncontrolled proliferation of basal cells leads to overcrowding of undifferentiated cells in the upper layers, while at the same time secretion of proteases causes remodeling of the matrix [49,50]. In advanced stages of dysplasia (Fig. 2c) severe remodeling of the ECM occurs, reflected in the alteration of flat architecture of ECTI by formation of rete ridges as epithelial cells start protruding downwards into the lamina propria without physically disrupting the BM [51]. Cytological features, such as pleomorphic and atypical cells and nuclei are also depicted in Fig. 2c under the grade of severe dysplasia. MPAM-SHGM cross-sections of imaged sites show similar features (Fig. 2d-f) to the model. MPAM autofluorescence from the keratinizing stratum corneum, the epithelium and lamina propria is shown in red, while SHGM from the fibrillar collagen network in the lamina propria is shown in green. Autofluorescence from the muscle beneath the ECM (Fig. 2d) (shown in red) and is comparable to the muscle seen in the deepmost layer visible in Fig. 2g. A faint green layer in the deep most portion of the MPAM-SHGM composite (Fig. 2d) arises from the lamina propria of the opposing side of the folded buccal pouch. The transition between epithelium (red) and ECM (green) is considered to represent the ECTI (white arrows in Fig. 2d-f), where many early dysplastic features are considered to be initiated [4,45,46]. Cross-sectional views of normal (Fig. 2d) and mild dysplastic (Fig. 2e) epithelium do not provide the contrast to visualize individual cell nuclei (epithelial cells are somewhat flattened in this state), but nuclei in the severe dysplasia become enlarged enough and polarized in a direction perpendicular to the surface to be easily visible (Fig. 2f, “*”). Thickening of the epithelial layer is also seen in mild and severe dysplasia compared to normal. Fig. 2g-i show H&E stained histology sections of the corresponding sites from Fig. 2d-f. Fig. 2g shows a normal hamster oral mucosa with characteristic features such as a thin keratinizing layer (~10 μm) at the top, followed by tightly packed epithelium and a densely packed ECM with the ECTI representing a linear interface (black arrows in Fig. 2g-i) separating ECM from the epithelium. Fig. 2h and 2i show features of mild and severe dysplasia respectively, such as epithelial thickening, abnormal variation in nuclear and cellular shape and size, large number of poorly differentiated cells, and irregular ECTI with rete-ridges. The features seen in Fig. 2g-i are similar to those revealed by MPAM-SHGM (Fig. 2d-f).

**Fig 3:**
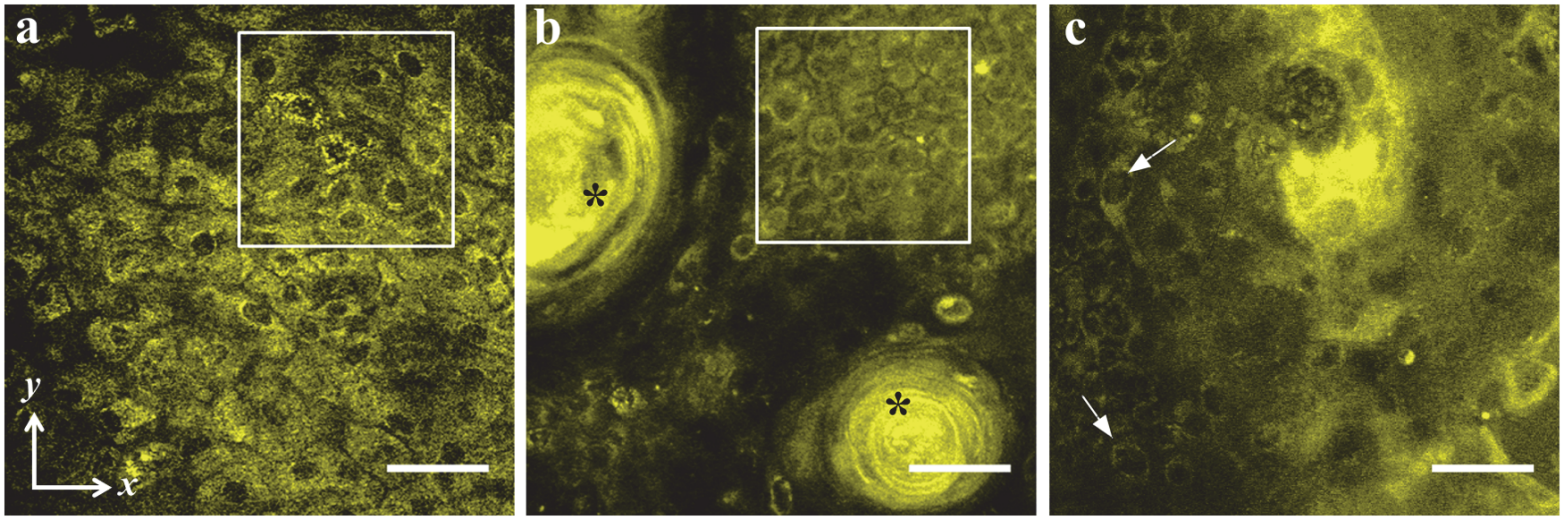
Cytological features visible in *in-vivo* MPAM are shown from normal (a), and dysplastic (b,c) epithelium of hamster oral mucosa. White boxes in “a” and “b” shows comparison of nuclear density between a normal (0.25 nuclei/100 μm^2^) and dysplastic epithelium (0.12 nuclei/100 μm^2^). “*” shows presence of keratin pearls (b) and arrows point towards enlarged nuclei (c) in dysplastic epithelium.

Fig. 3 shows single x-y micrographs of superficial epithelium from a normal (Fig. 3a) hamster mucosa compared against representative dysplastic mucosa (Fig. 3b and 3c) at a similar depth of 20–25 μm. In the normal mucosa the epithelium is seen as a regular mosaic pattern of superficial epithelial cells having dark nuclei (diameter: 8–10 μm) and highly autofluorescent cytoplasm (Fig. 3a). There was also no abnormal variation of size or shape within the nuclei. Whereas in dysplastic epithelium an overcrowding of immature cells with abnormal keratin production seen as keratin pearls (Fig. 3b, denoted by “*”) and recognized as features of premalignant changes can be visualized. Abnormally large nuclei (diameter: 16–18 μm) representing anisonucleosis can be seen in Fig. 3c (white arrows). Areas enclosed with white squares show a higher nuclear density in dysplasia (0.25 nuclei/100 μm^2^) than normal (0.12 nuclei/100 μm^2^). These cytological features of dysplasia are well established and results are comparable to those reported elsewhere [25,37,52].

**Fig 4:**
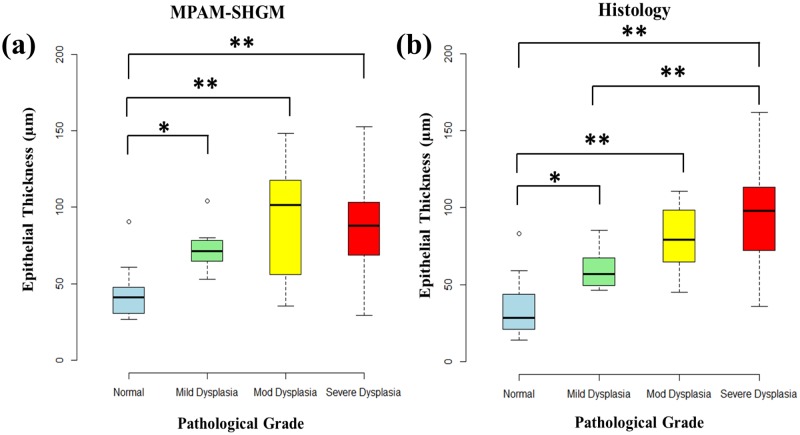
Epithelial Thickness measurements from MPAM-SHGM (a) and histology (b) show statistically significant increase in thickness of mild, moderate and severe dysplasia from normal mucosa. Box plot analyses show the median, 25^th^ and 75^th^ percentiles, and outliers for both MPAM-SHGM and histology. “*” and “**” represent statistical difference between groups at p<0.05 and 0.01 significance respectively.

Fig. 4 shows a comparison of epithelial thickness measurements by MPAM-SHGM (Fig. 4a) and histology (Fig. 4b). Box plots show the distribution of median epithelial thickness for normal, mild, moderate and severe dysplasia. Statistical analysis by single factor ANOVA depicts that the differences in epithelial thickness between normal and all grades of dysplasia are highly significant (shown by “*”) by both MPAM-SHGM and histology. The average epithelial thicknesses of all the dysplasia groups are higher than normal tissue and they show a similar trend in both MPAM-SHGM and histology except moderate dysplasia in MPAM-SHGM has a larger median and standard deviation. Larger standard deviations in higher grades of dysplasia (moderate and severe) are consistent with the observation that epithelial thicknesses in higher grades of dysplasia are not homogeneous and display a large variation compared to normal and mild dysplasia.

**Fig 5:**
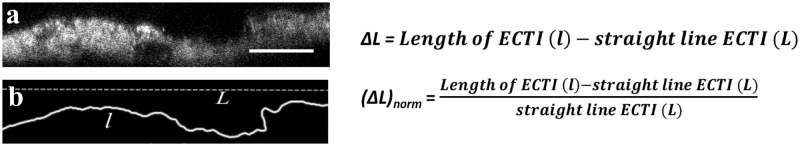
Cross-sectional x-z SHGM micrograph is shown in (a). Figure (b) shows an ECTI (solid white line) extracted from (a) after the FeatureJ: Edges plugin in ImageJ was applied. The dashed line represents the reference linear distance between the two ends of the image. The equations on the right were used to calculate ΔLinearity (ΔL_norm_). Scale bar: 50 μm.

**Fig 6:**
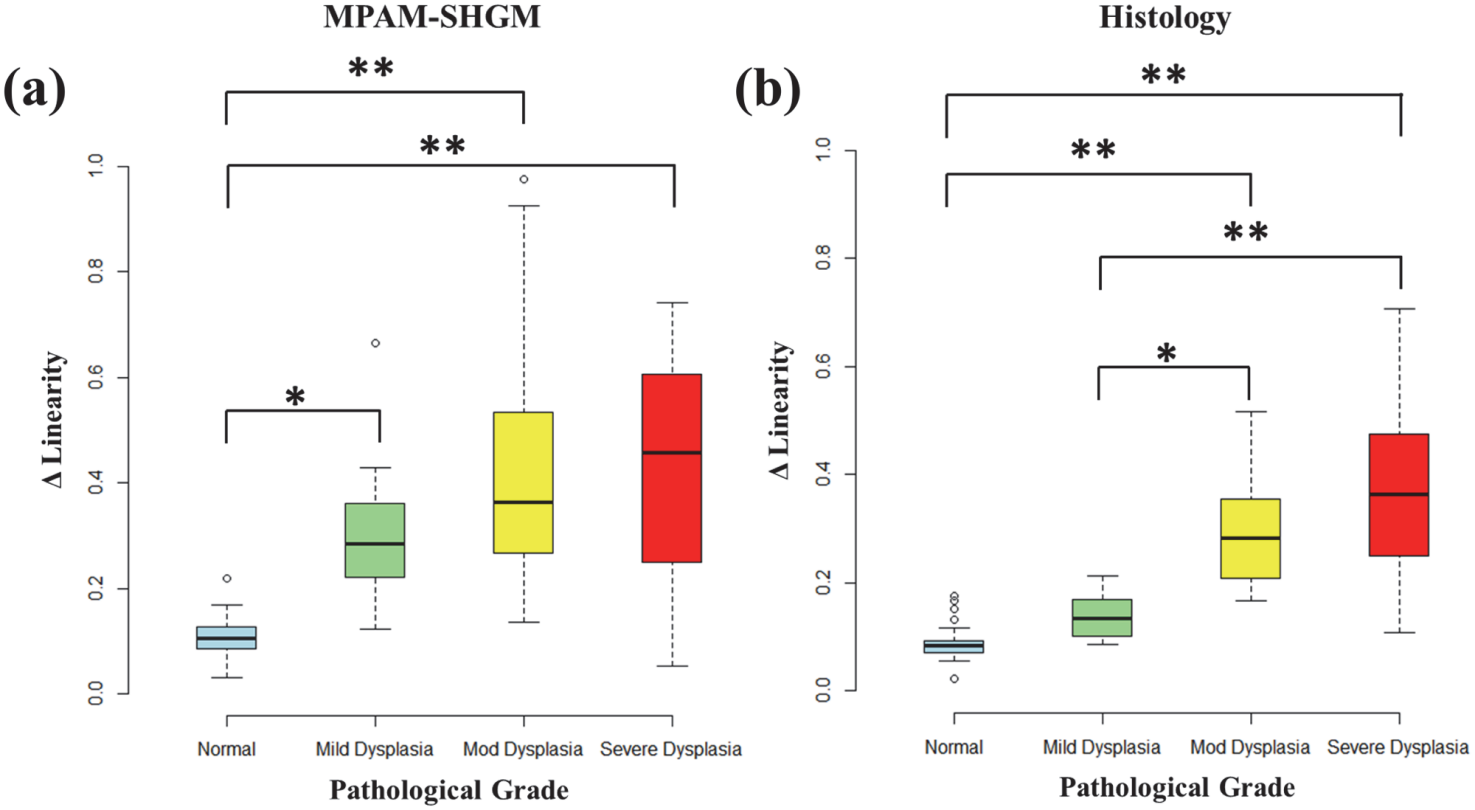
ΔL_norm_ measurements from MPAM-SHGM (a) and histology (b) show statistically significant increase in ΔLinearity of mild, moderate and severe dysplasia from normal mucosa. Box plot analyses show the median, 25^th^ and 75^th^ percentiles, and outliers for both MPAM-SHGM and histology. “*” and “**” represent statistical difference between groups at p< 0.05 and 0.01 significance respectively.

Fig. 5 describes the analysis of the ECTI ΔLinearity from SHGM cross-sections. The SHG signal from a moderately dysplastic epithelium is shown in cross-section in Fig. 5a with clearly visible deformations of the ECTI. Fig. 5b shows the ECTI after semi-automated segmentation, as described in Materials and Methods, of the SHGM cross-section and the equations describe the approach taken for calculation of ΔLinearity. Thirty-three normal sites collected from mineral oil treated and DMBA treated hamsters and twenty-seven sites with dysplasia collected from DMBA treated hamsters were analyzed. H&E stained histology images and SHGM images from each site were analyzed for ECTI irregularity (Fig. 6), represented as ‘ΔLinearity.’ All dysplastic groups showed significantly higher ΔLinearity than normal ECTI for both MPAM-SHGM (Fig. 6a) and histology (Fig. 6b) except mild dysplasia in histology, possibly due to a large sampling area in biopsy than microscopy which was approximately twice the size as assurance that the imaged region was sampled by histology. The distributions of ΔLinearity of moderate and severe dysplasia were larger than normal and mild dysplasia similar to epithelial thickness. Average ΔLinearity for normal and all dysplastic grades in MPAM-SHGM images were calculated to be 0.11±0.04 for normal, 0.31±0.17 for mild dysplasia, 0.45±0.29 for moderate dysplasia and 0.41±0.22 for severe dysplasia. Similar calculations on histology images revealed ECTI ΔLinearity of 0.09±0.03 for normal, 0.14±0.04 mild dysplasia, 0.29±0.1 for moderate dysplasia, and 0.38±0.19 for severe dysplasia. Average ΔLinearity of dysplastic and normal tissue from MPAM-SHGM and histology are shown in Table 1.

**Table 1:** Summary of the statistical analyses performed from ΔLinearity measurements carried out on cross-section images from MPAM-SHGM or histopathology.

Group		Avg. ΔLinearity (SD)	AUC	Sensitivity (%)	Specificity (%)	TP	FP	TN	FN
MPAM-SHGM	Normal	0.11 (0.04)	0.95	87.9	97.6	0.7	0.3	0.96	0.04
	Dysplasia	0.41 (0.24)							
H&E	Normal	0.09 (0.03)	0.96	96.4	85.7	0.59	0.41	0.95	0.05
	Dysplasia	0.28 (0.16)							

SHGM: Second Harmonic generation Microscopy, SD: Standard Deviation, AUC: Area Under the Curve, TP: True Positive, FP: False Positive, TN: True Negative, FN: False Negative.

**Fig 7:**
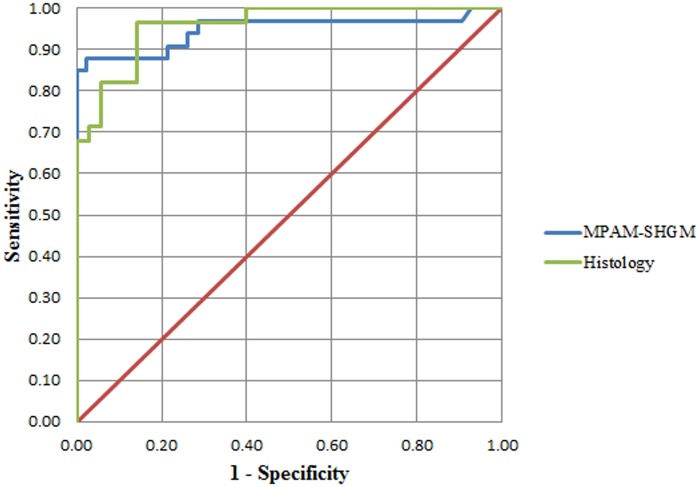
ROC curve at 95% confidence for ΔLinearity calculated from MPAM-SHGM (Blue) and histological (Green) images.

As we believe the ECTI shape changes are a direct consequence of interactions between neoplastic cells, the basement membrane and ECM of the lamina propria that occur with dysplasia, ROC analysis was performed to test the ability of ΔLinearity to differentiate normal from dysplasia. ROC curves for MPAM-SHGM and histology were analyzed for ΔLinearity for normal vs. dysplasia (mild, moderated, severe) (Fig. 7). An ROC plot represents true positive rate against false positive rate for different possible cut-offs of ΔLinearity calculated either from histopathology (Fig. 7, green line) or from microscopy (Fig. 7, blue line). Table 1 summarizes the results from ROC analysis. As can be seen in Fig. 7 the ROC curves for MPAM-SHGM and histology are very similar and they produced comparable area under the curve (AUC), which is a measure of accuracy of a test. AUC values from ΔLinearity ROC for both MPAM-SHGM and histology are greater than 0.9, considered a successful test. Table 1 shows the sensitivity and specificity of ΔLinearity for MPAM-SHGM and histology respectively. True positives, false positives, true negatives and false negatives are also shown in Table 1 for comparison between MPAM-SHGM and histology.

## Discussion

The current study was undertaken to demonstrate a new noninvasive way to visualize and characterize the ECTI during dysplasia when other well characterized early neoplastic changes in the epithelium are known to occur. Previously published studies have indicated NLOM by either MPAM or SHGM may provide a noninvasive method to identify sites of dysplasia in oral epithelium through assessment of either cytologic or ECM features [22,37,52–55]. We demonstrated the ability to noninvasively and quantitatively evaluate ECTI *in-vivo*, providing a new way to study early epithelial dysplastic changes using a hamster model of oral cancer/dysplasia. The hamster model provided the ability for noninvasive *in-vivo* imaging in a buccal mucosa in which the ECTI is relatively flat (Fig. 2a, d, and g), similar to the human buccal mucosa, floor of the mouth and the ventral surface of the tongue in human oral cavity [34], all common sites for neoplastic transformation. Our interest in ECTI shape is based on the potential central role this interface serves in neoplastic transformation. There are indications that early pathological events leading to neoplastic transformation of basal epithelium initiate structural alterations in the ECTI [56]. Neoplastic epithelial cell proliferation leads to non-uniform expansion of the epithelium resulting in focal increases in epithelial thickness and shape changes to the ECTI, including formation of rete ridges [4,6,34]. Invasion is initiated when the BM is disrupted and neoplastic epithelial cells migrate into the underlying lamina propria, crossing the BM/ECTI. Thus, given the potential role the ECTI may play, the presented MPAM-SHGM method to provide noninvasive qualitative and quantitative assessment of the ECTI could be valuable in studies of epithelial neoplasia, particularly if coupled with other known parameters associated with precancer and cancer development.

Studies resulting in direct assessment of irregularities in ECTI morphology have been restricted to histological evaluation and in some part electron microscopy [1,14,15,23,37,57,58]. Studies have been reported in which density [34] or shape [58] of rete ridges from histological sections under normal and dysplastic conditions were quantified. Others have demonstrated ECTI irregularities in histology by looking at tissue complexity based on fractal geometry calculations [1,4]. Examination of the ECTI by electron microscopy also revealed irregular ECTI with discontinuities and prominent focal thickening of the BM during epithelial dysplasia and invasive carcinoma [14,15]. All of these studies required tissue removal and extensive processing of the excised tissue to extract ECTI parameters.

A key advantage of the method presented in the current study is that it overcomes the need for the series of sample preparation steps needed in the traditional methods of histology and electron microscopy such as tissue resection, fixation, and labeling/staining. The current method allows direct imaging of the ECTI without disruption to the epithelium and requires no exogenous stains. Further, delineation of the ECTI is without ambiguity as there is no SHG signal in the epithelium, arising only from noncentrosymmetric molecules in the lamina propria (fibrillar collagen), and creating a ‘hard’ interface defining the transition from a region of no SHG signal (epithelium) to one with strong SHG signal (lamina propria). Corresponding MPAM based on autofluorescence provides the ability to visualize cells throughout the full epithelium which then transitions into a fibrous network in the lamina propria as demonstrated in the current study and in previous studies [24,25,52,53]. In two-color cross-sectional overlays of MPAM-SHGM combining the two sources of contrast, the epithelium, lamina propria, and ECTI are readily identified (Fig. 2d-f).

While straightforward delineation of the ECTI with MPAM-SHGM is highly advantageous, we emphasize that a key point of this study is not only visualization of the ECTI but introduction of a shape parameter to describe linearity which was indicative of abnormality. As discussed above ECTI shape changes are known to occur with dysplasia and are likely based on direct events in cell proliferation and ECM remodeling among other factors during transformation from a normal mucosa. Based on the observation that the ECTI shape went from relatively flat to curved, forming rete-ridges (Fig. 2: arrows) with dysplasia, we introduced a parameter representing deviation of ECTI from absolute linearity (ΔLinearity). With more rete ridges present in the dysplastic samples, the average ΔLinearity between two given points on the ECTI surface was expected to increase relative to a flat appearance observed in normal mucosa. Results did in fact show a significantly higher ΔLinearity in dysplasia relative to normal seen both by noninvasive imaging and in corresponding histology (Fig. 6). ROC analysis showed the potential of ΔLinearity to differentiate dysplastic abnormality from normal mucosa with AUC and sensitivity/ specificity comparable to analysis based on H&E (Fig. 7). We conclude that the method of SHGM was comparable to histopathological assessment for differentiating dysplasia from normal mucosa.

The same processes that lead to alterations in ECTI morphometry result in an increase in epithelial thickness, and as would be expected, a statistically significant increase in epithelial thickness was measured with grade of dysplasia (Fig. 4). Distribution of thickness measurements for moderate and severe dysplasia was larger than normal and mild dysplasia in both MPAM-SHGM and histology due to heterogeneity (non-uniform thickness) that results at the lesion site. Highest agreement between MPAM-SHGM and histology occurred for both normal and severe dysplasia. Differences in epithelial thickness and linearity between imaging and histology for mild and moderate dysplasia may be attributed to the larger sampling area required for biopsy compared to the imaging lateral field-of-view. Both methods (imaging and biopsy) were centered over the most central part of the lesion which generally comprised the most severe pathology relative to the periphery. Histological analysis was restricted to the most central part of the lesion but sampled over approximately twice the lateral range for assurance that the imaged site was included. Thus, in cases of smaller lesions MPAM-SHGM likely fully comprised the most abnormal part of the lesion whereas in a histological section less severe areas from the periphery could have also been included (e.g. mild dysplasia). The result may have been higher heterogeneity in epithelial thickness and ECTI being more prominent in imaging than histology. Nonetheless, imaging resulted in similar trends to histology and alterations in epithelial thickness during dysplasia were consistent with those previously reported by other methods [52,59,60]. Cellular atypia was also observed as expected and consistent with the use of MPAM imaging in epithelial dysplasia [13,52,61].

The observed cellular and thickness measures supports the use of MPAM-SHGM for assessing both the newly described ECTI parameter and other previously described (cellular, epithelial) parameters. ΔLinearity introduced in this study could be considered an additional marker of abnormality that could be used to quickly identify sites that may contain abnormalities when imaging by NLOM or quantitatively combined with cytological, layer based, and matrix features to detect/stage sites of dysplasia. With the number of sites evaluated in the current study, such multi-parameter assessments could not be performed, but is something that should be explored in future work.

An exciting application of the method introduced in this study is in longitudinal studies to provide a better understanding of the dynamic interaction between epithelium and lamina propria through the ECTI in early dysplasia and perhaps early cancer. The noninvasive nature of the method is ideal for longitudinal studies requiring repeated measures. Few methods provide this possibility coupled with high resolution assessment that can simultaneously provide subcellular imaging with MPAM or even molecular imaging through the use of targeted fluorophores. Histological assessment of ECTI in surgical biopsies is limited due to tissue shrinkage and distortions after incision while *in-vivo* MPAM-SHGM method provides ECTI features in its native environment. Stem cell studies have shown potential for clinical use in recent times where the resistance to radio- and chemotherapies, and tumor recurrence has been attributed to cancer stem cells in the basal epithelium [62]. Molecular markers such as CD44H [63], p75 [64], K15 [62] were identified as oral stem cell markers that also play important roles in development of dysplasia and OSCC. For examination of the role of stem cells, for example, ΔLinearity computed from ECTI could be coupled to imaging of fluorescently labeled stem cells which could provide new avenues for *in-vivo* monitoring of neoplastic transformation and study the relationship between abnormalities in basal epithelium and ECTI dynamics.

Finally, it is noted that the linearity shape parameter developed in this study is one that has potential to be applied to other noninvasive imaging modalities in which the ECTI is resolved/delineated. Such an example may be in OCT, which provides cross-sectional views of oral mucosa in a noninvasive manner [16–21]. The epithelium-to-lamina propria transition is more subtle in OCT than the ‘hard’ interface provided by SHG but separation of the two layers is possible. At least two studies have demonstrated segmentation of OCT cross-sectional images (B-scans) using advanced image processing to show separation of the epithelium from the lamina propria [20,21], however thus far direct quantitative comparison of this interface with the BM or ECTI in histology has not been performed, nor has the ECTI morphometry been described in neoplasia using OCT to the best of our current knowledge.

In summary, the current study demonstrated an *in-vivo* imaging approach to identify early indication of ECTI alteration using MPAM-SHGM imaging. To our knowledge this is the first study to examine and quantify features of ECTI using noninvasive optical microscopy. The ability to detect and differentiate early changes in ECTI in a preclinical model provides a powerful way to study processes of early neoplasia and could be developed for imaging of other epithelial sites as well as in human epithelium. As nonlinear optical microscopy approach depths beyond 1 mm [65–68] potential clinical application has started to look promising and with further development improved diagnostic information is expected.
